# Diagnostic Performance of Extracellular Volume Quantified by Dual-Layer Dual-Energy CT for Detection of Acute Myocarditis

**DOI:** 10.3390/jcm10153286

**Published:** 2021-07-26

**Authors:** Salim Aymeric Si-Mohamed, Lauria Marie Restier, Arthur Branchu, Sara Boccalini, Anaelle Congi, Arthur Ziegler, Danka Tomasevic, Thomas Bochaton, Loic Boussel, Philippe Charles Douek

**Affiliations:** 1 Department of INSA-Lyon, University of Lyon, University Claude-Bernard Lyon 1, UJM-Saint-Étienne, CNRS, Inserm, CREATIS UMR 5220, U1206, 69621 Lyon, France; sara.boccalini@chu-lyon.fr (S.B.); loic.boussel@chu-lyon.fr (L.B.); philippe.douek@chu-lyon.fr (P.C.D.); 2 Cardiovascular and Thoracic Radiology Department, Hospices Civils de Lyon, 69500 Lyon, France; arthur.branchu@chu-lyon.fr (A.B.); arthur.ziegler69@gmail.com (A.Z.); 3 Rockfeller Faculty of Medicine, Lyon Est, University Claude-Bernard Lyon 1, 69003 Lyon, France; lauria.restier@etu.univ-lyon1.fr (L.M.R.); anaelle.congi@etu.univ-lyon1.fr (A.C.); 4 Department of Cardiology, Louis Pradel Hospital, Hospices Civils de Lyon, 59 Boulevard Pinel, 69500 Bron, France; danka.tomasevic@chu-lyon.fr (D.T.); thomas.bochaton@chu-lyon.fr (T.B.)

**Keywords:** dual-energy CT, iodine, diagnostic imaging, myocarditis, extra-cellular volume

## Abstract

Background: Myocardial extracellular volume (ECV) is a marker of the myocarditis inflammation burden and can be used for acute myocarditis diagnosis. Dual-energy computed tomography (DECT) enables its quantification with high concordance with cardiac magnetic resonance (CMR). Purpose: To investigate the diagnostic performance of myocardial ECV quantified on a cardiac dual-layer DECT in a population of patients with suspected myocarditis, in comparison to CMR. Methods: 78 patients were included in this retrospective monocenter study, 60 were diagnosed with acute myocarditis and 18 patients were considered as a control population, based on the 2009 Lake and Louise criteria. All subjects underwent a cardiac DECT in acute phase consisted in an arterial phase followed by a late iodine enhancement phase at 10 min after injection (1.2 mL/kg, iodinated contrast agent). ECV was calculated using the hematocrit level measured the day of DECT examinations. Non-parametric analyses have been used to test the differences between groups and the correlations between the variables. A ROC curve has been used to identify the optimal ECV cut-off discriminating value allowing the detection of acute myocarditis cases. A *p* value < 0.05 has been considered as significant. Results: The mean ECV was significantly higher (*p* < 0.001) for the myocarditis group compared to the control (34.18 ± 0.43 vs. 30.04 ± 0.53%). A cut-off value of ECV = 31.60% (ROC AUC = 0.835, *p* < 0.001) allows to discriminate the myocarditis with a sensitivity of 80% and a specificity of 78% (positive predictive value = 92.3%, negative predictive value = 53.8% and accuracy = 79.5%). Conclusion: Myocardial ECV enabled by DECT allows to diagnose the acute myocarditis with a cut-off at 31.60% for a sensitivity of 80% and specificity of 78%.

## 1. Introduction 

Affecting approximately 1.8 million people worldwide in 2017 [1], myocarditis is a frequent inflammatory disease of the heart that can be caused by infectious agents, exposure to toxic substances and immune system activation [2,3]. The diagnosis includes clinical, laboratory, imaging, and histological parameters [1]; so over the years, different diagnostic tests have been developed to identify patients that have acute myocarditis. The endomyocardial biopsy has been the gold standard for a while until cardiac magnetic resonance imaging (CMR) and computed tomography (CT) got considered as non-invasive alternatives [4,5]. CMR emerged as a powerful non-invasive method for tissue characterization, including recognition and quantification of inflammation and replacement fibrosis in the setting of acute myocarditis [6,7]. Thanks to the Lake Louise Criteria, published in 2009, three markers of myocardial inflammation have been identified: hyperemia, tissue edema and necrosis/fibrosis [7]. Among the markers of the inflammatory burden, one of them stands out from the crowd: the extracellular volume (ECV).

Myocardial ECV increases related to myocardial fibroses, cardiac amyloid or edema [8]. CMR is the reference to measure ECV, but some previous studies showed that ECV can also be successfully determined with computed tomography, whether with single-energy computed tomography (SECT) or with dual-energy computed tomography (DECT), with a high correlation between ECV measurements derived from CT, histologic quantification, and CMR [9,10,11,12,13,14]. Hence, CT can effectively be considered as an interesting alternative to CMR. In addition, CT is more accessible and cheaper, while having a faster acquisition with a better spatial resolution, which makes it a good candidate for cardiac emergency imaging [15]. 

Therefore, we investigated the diagnostic performance of myocardial ECV quantified by cardiac DECT in a population of patients with suspected myocarditis, in comparison to CMR. 

## 2. Materials and Methods

### 2.1. Study Design 

This study is a monocenter retrospective study which has been conducted in the Cardiologic Hospital Louis Pradel, in Lyon (FRANCE) from May 2018 to May 2021. 

### 2.2. Population 

The population was constituted of two groups of patients that were addressed for suspicion of acute myocarditis and underwent a cardiac dual-energy computed tomography and a CMR at the acute phase: patients with confirmed acute myocarditis (MG = myocarditis group) and patients without myocarditis patterns (CG = control group). The diagnosis was confirmed according to the Lake and Louise criteria on CMR. In order to validate the diagnosis of myocarditis, the patient had to present at least two of the Lake Louise Criteria (i.e., hyperemia, tissue edema and/or fibrosis/necrosis) [7]. Exclusion criteria for the myocarditis group in order to be comparable to the control group were: an underlying cardiomyopathy and/or left ventricular ejection fraction (LVEF) ≤40% and/or acute cardiac complications (heart failure, life-threatening arrythmias, death). Exclusion criteria for the control group were: an underlying cardiomyopathy and/or LVEF <50% according to the definition of heart failure [16].

### 2.3. Data Registration

The following clinical, biological, functional and imaging parameters were recorded at admission: (1) age, sex, weight, size, systolic and diastolic blood pressure (SBP, DBP), heart rate, (2) values of high-sensitivity troponin (Tn), brain natriuretic peptide (BNP), creatinine; (3) left ventricular ejection fraction (LVEF) on trans-thoracic ultrasound at admission.

### 2.4. DECT Imaging Protocol

#### 2.4.1. Injection Protocol

The contrast material used was Iomeprol (400 mg/mL, Iomeron^®^; Bracco, Milan, Italy). All patients underwent a standard coronary CT angiography injection protocol consisting on a bolus injected at 3.5 mL/s into an 18 G catheter. The injection material was followed up by a saline rinse of 20 mL. The bolus volume was calculated according to the weight of the patient (1.2 mL/kg of contrast material).

#### 2.4.2. Image Acquisition

The examinations have been performed on a dual-layer dual-energy CT (iQon; Phillips, Haifa, Israel) consisting of a first pass arterial phase and late phase 10 min after injection. Acquisition parameters were a retrospective gated ECG acquisition at 120 kVp, with a cardiac care mas dose modulation (full dose 78%, half dose 40%), a pitch value at 0.20, and a rotation time at 0.27 s. Further technical details are provided in previous studies [17,18,19].

#### 2.4.3. Reconstruction Protocol

Conventional and iodine density images were reconstructed from the late cardiac acquisition with a 1.5 mm slice thickness, a standard filter (Filter B) and a large field-of-view at 500 mm. 

#### 2.4.4. Image Analysis

Images were analyzed using a clinical workstation (Spectral Phillips Intellispace Portal Station, Phillips; Haifa, Israel). The myocardium was analyzed and manually segmented in 16 AHA segments using this software, on the iodine density images. We extracted the iodine concentrations in mg/mL for each 16 AHA segments. A circular region-of-interest of ~530 mm^2^ was drawn in the left cardiac cavity for measuring the iodine concentration in blood. The extracellular volume (ECV) was then calculated such as following:

(1) $$\mathrm{ECV}=100\times \left(1-\mathrm{Ht}\right)\times \frac{\left(\mathrm{Iodine}\;\mathrm{concentration}\;\mathrm{in}\;\mathrm{the}\;\mathrm{myocardium}\right)}{\left(\mathrm{Iodine}\;\mathrm{concentration}\;\mathrm{in}\;\mathrm{the}\;\mathrm{blood}\right)}$$

The iodine concentration was measured on iodine images in mg/mL; “Ht” is the hematocrit measured the day of the DECT acquisition. From these measurements, we analyzed the global myocardial ECV-per-patient. 

### 2.5. Radiation Dose

Dose-length-product and volume CT dose index for the late cardiac acquisition were recorded. Mean effective dose was calculated multiplying the dose-length-product by the chest k-factor of 0.014 [20].

### 2.6. Statistical Analyses 

Statistical analyses were performed with the SPSS software (IBM SPSS Statistics 19; 2018). The data are expressed as mean ± MSE (mean standard error) and median (minimum-maximum), accordingly to the normality tests. 

For comparison of continuous variables between the two study groups, a non-parametric Mann–Whitney test has been used. A non-parametric Kolmogorov–Smirnov test has been used for the nominal variables. 

For correlation purposes, Spearman correlation coefficients using a 95% confidence interval were calculated between biological (BNP, creatinine), functional markers (LVEF) and the global myocardial ECV tested. 

A ROC curve has been used to identify the best discriminative cut-off value of ECV and to calculate the sensitivity, the specificity, the positive predictive value, the negative predictive value and the accuracy for the diagnosis of myocarditis, after ranking all the values and linking each value to the diagnosis of myocarditis.

## 3. Results

### 3.1. Characteristics of the Population 

**Figure 1:**
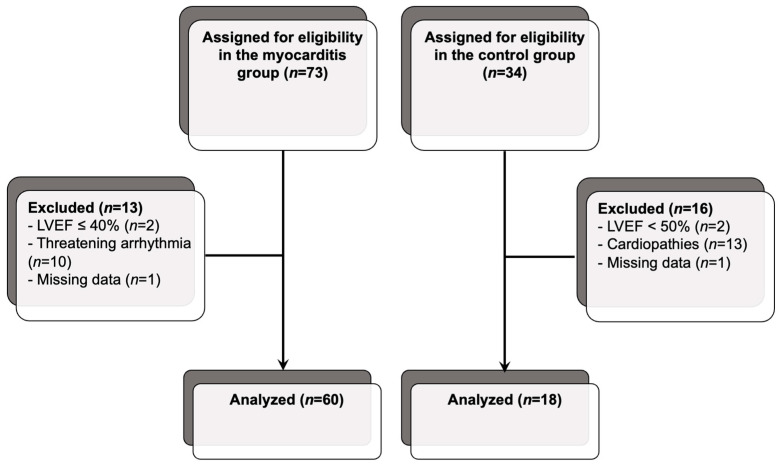
Flow chart of the study population.

Initially, 107 patients were addressed to the hospital for suspicion of acute myocarditis. A total 73 patients have been diagnosed with acute myocarditis while 34 patients were rules out from myocarditis on CMR. Among them, 13 patients were excluded: 2 patients had a left ventricular ejection fraction ≤ 40%, 10 patients had life-threatening arrhythmia, and one patient was missing data. Finally, 60 patients have been included in the acute myocarditis group (Figure 1). Concerning the control group (CG), only 18 subjects have been included. Among the 16 patients who have not be retained for the CG, 2 of them had a left ventricular ejection fraction < 55%, 13 had cardiomyopathies and one patient had missing data (Figure 1). The baseline characteristics of the study population are summarized in Table 1. 

The patients of the two groups have been paired by age and sex. The mean age was 32.9 ± 1.4 years for the (MG) and respectively 35.1 ± 3.6 years for the (CG). No significant difference for mean age or male proportion has been found between the two study groups. When compared, the troponins measured level was significantly higher (*p* < 0.001; 8630.3 ± 1585.9 vs. 822.5 ± 339.9 ng/L), and the LVEF was significantly lower (*p* = 0.006; 57.6 ± 1.0 vs. 64.2 ± 1.7%) for the acute myocarditis group compared to the control group.

### 3.2. Measurement of the Myocardial Inflammation 

**Figure 2:**
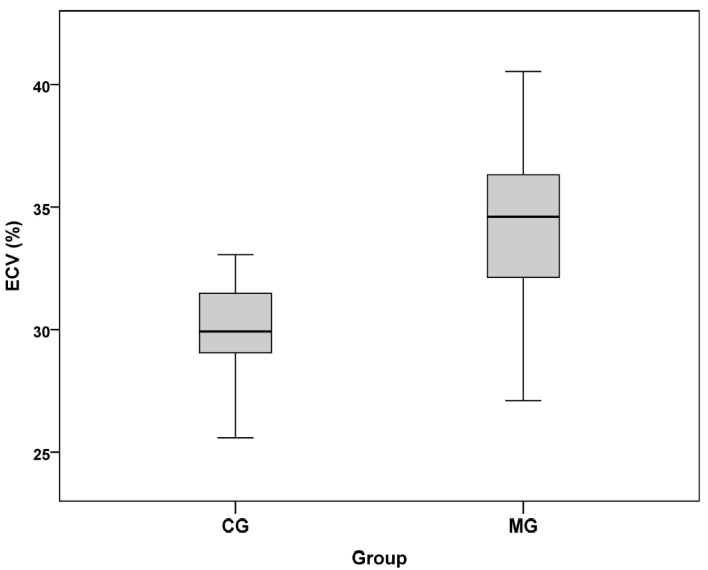
Box plots of the mean ECV for both groups. Median is represented by the line and the mean by the cross in the center of the box, upper and lower margins correspond to the 25th and the 75th percentiles, and outliers indicate the minimal and the maximal values. ECV = extracellular volume; CG = control group; MG = myocarditis group.

Mean ECV of the myocarditis group was significantly higher compared to the one measured for the control group (*p* < 0.001, CI 95% (28.9–31.2)). Results are summarized in Table 2 and in Figure 2. 

### 3.3. Correlation between ECV and the Different Parameters

Concerning the acute myocarditis group, a positive significant correlation (*p* = 0.011) has been found between ECV and the troponins level (Pearson coefficient = 0.325). No significant correlation between ECV and LVEF has been found for this group. 

On the other hand, significant correlations (respectively *p* = 0.015 and *p* = 0.048) have been found between ECV and the weight (Pearson coefficient = −0.563) and the BMI (Pearson coefficient = −0.472). No significant correlation has been found between ECV and the troponins level for this group. All the results are summarized in Table 3.

### 3.4. Measurement of the ECV Cut-Off Value 

**Figure 3:**
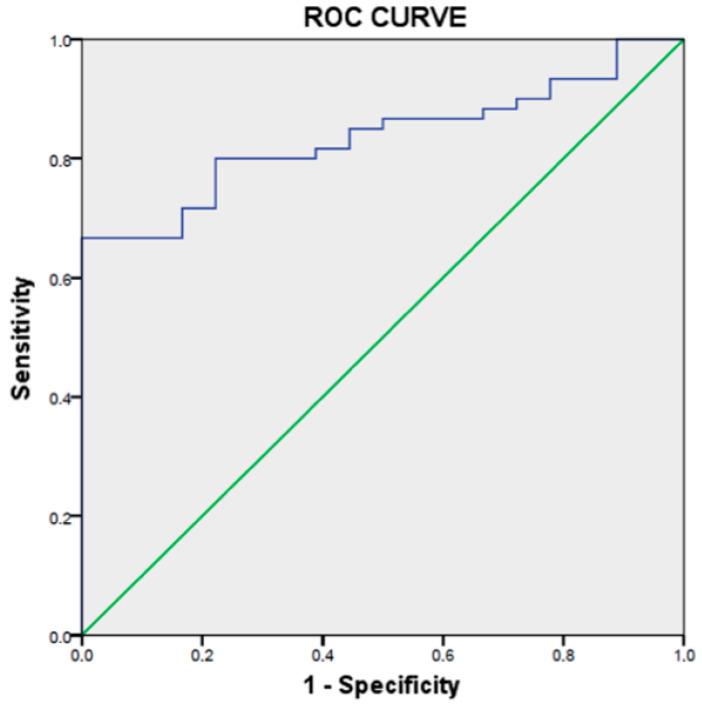
ROC curve representing the different cuts-off of ECV.

**Figure 4:**
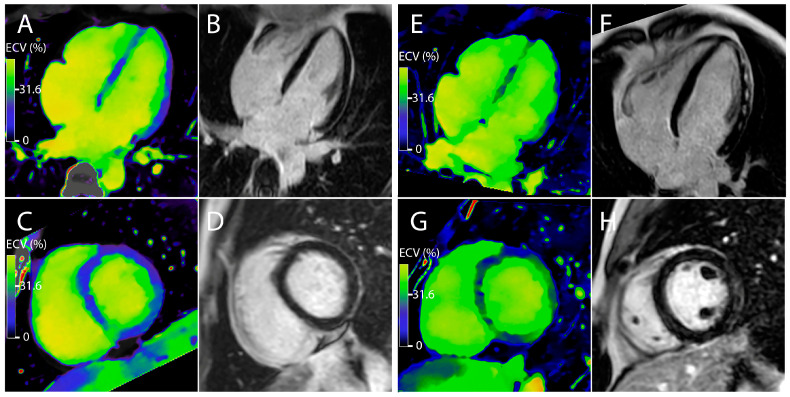
Representative cases of patients with suspected acute myocarditis. Left panel (**A**–**D**). Case of a 42-year-old woman with elevated high-sensitivity troponins at 83 ng/L at admission. A late phase DECT was performed at admission. The global ECV was measured at 29.7%. MRI did not show any late gadolinium enhancement. Right panel (**E**–**H**). Case of a 27-year-old man with elevated high-sensitivity troponins at 8000 ng/L at admission. A late phase DECT was performed at admission. The global ECV was measured at 35.1%. MRI showed multiple late gadolinium enhancement of the sub-epicardial myocardial wall in favor of myocarditis.

A ROC curve has been realized to estimate the best ECV cut-off value to finally determine which value can be used to discriminate acute myocarditis in a group of patients with suspected myocarditis. This curve is significantly representative of the ECV cuts-off values (*p* < 0.001) with an area under the curve of 0.835 (with a 95% CI of (0.748–0.922)). The results are represented in the Figure 3 and Figure 4. The retained cut-off value of 31.60% in our study has shown a sensitivity of 80%, a specificity of 78%, a positive predictive value of 92.3%, a negative predictive value of 53.8% and accuracy of 79.5% for the discrimination of acute myocarditis from the control subjects.

### 3.5. Radiation Dose Analysis

The mean ± SD volume CT dose index was 6.2 ± 1.7 mGy, the total dose length product was 123.2 ± 39.6 mGy.cm^−1^. As a result, the mean ± SD equivalent dose was calculated to be 1.7 ± 0.5 mSv. 

## 4. Discussion

In the present study, we showed that myocardial ECV quantified by DECT is a biomarker of the myocarditis burden and that can be used for discriminating acute myocarditis in a population of patients with suspected myocarditis. These findings support the diagnostic value of ECV in the diagnostic work-up of a suspected acute myocarditis.

The novelty of the present study is the use of CT in a suspected population of myocarditis and as so the report of ECV values which can be the starting point for its implementation in clinical routine. In the same line, few studies have reported the ECV values of different cardiopathies such as heart failure, global cardiomyopathies, cardiac amyloidosis or in aortic stenosis patients [14,21,22]. Taken together, these studies are holding great promises for cardiac CT imaging because of its many advantages. Cardiac CT allows a 3D registration along the heart muscle in a short time acquisition with an excellent spatial resolution, and direct measurement of ECV in opposition with CMR which relies on measuring the effect of GBCAs on protons [8]. Because of its poor availability and its numerous contraindications, CT seems to be an encouraging and interesting alternative to CMR despite its irradiation [23]. However, among the different CT systems, it has to be noted the great advantage for DECT technology that allows the measurement of iodine content in a tissue without requiring a pre-injection examination such as done with single-energy CT, which reduces the burden radiation dose [22,24,25,26]. By decomposing the X-ray spectrum in two different energies spectra, DECT systems measure the photoelectric and Compton effects. Their recombination will allow to reconstruct quantitative images of the iodine distribution in the myocardium for ECV measurement [24]. Hence, DECT imaging is more prone to cardiac tissue characterization than single-energy CT which opens the door to evaluation of the myocardial ECV. 

Our results demonstrated a significant increase of ECV in acute myocarditis patients, in accordance with previous studies using CMR that underlined an elevation of this biomarker in different cardiomyopathies, including myocarditis [15,27]. We observed significant correlation between ECV and both troponins and BNP, which is coherent with the physiopathology of myocarditis. This is explained by the fact that troponins are a marker of tissue inflammation damages and BNP of a myocardial stress [7]. While for the control group, which presented a low elevation of troponins—no correlation was found. One hypothesis would be the non-flawless performances of current DECT systems for quantification of low iodine concentrations, which are reflecting low ECVs [17]. Finally, we observed a significant association between ECV and presence of myocarditis with an AUC of 83.5% (*p* < 0.0001). We have determined a cut-off value of ECV of 31.60% with high sensitivity of 80% and specificity of 78% for discriminating myocarditis. These performances are similar to different CMR studies [28,29,30,31,32] with pooled performances reported in a meta-analysis with a sensitivity of 76%, a specificity of 76%, a PPV of 72%, a NPV of 79% [33]. In addition, Nadjiri et al. have proposed an optimal ECV cut-off of 32.4% with a sensitivity of 93% and specificity of 74%, using CMR [28]. Altogether, the ECV values reported in the present study are in line with the data available with CMR. This is not a surprising finding, considering the high concordance between these modalities for ECV quantification as demonstrated by recent comparative studies which permits the use of this biomarker for multiple prospects [8,11,14,21]. Hence, we showed recently that ECV enabled by DECT allows a prediction of cardiac complications in acute myocarditis [34]. In this recent study, the cut-off suggested was of 39.5% which is highlighting a higher myocarditis inflammation burden that is consequently at risk. Finally, the present study is bringing one more contribution to the DECT for being a quick appropriate alternative candidate to CMR in cardiac emergency facilities [23,35].

The present study has limitations. The main limitation relies on the unperfect sensitivity of CMR using the 2009 CMR Lake Louise criteria which do not take into account ECV [7]. This limitation is mainly explained by the retrospective design of the study that started before the revised criteria in 2018 [6]. This bias probably increases ECV in the control group via false negative patients. In consequence differences between groups is probably underestimated and the bias non-differential. The absence of a fair comparison of ECV with CMR is also a limitation that points out the availability issue of CMR in acute settings reflecting a real-world practice. Yet a recent study has demonstrated similar ECV between the DECT system used in the present study and CMR, reinforcing our confidence in ECV derived from DECT scans [36]. Finally, the incremental diagnostic value of ECV by DECT to the late iodine enhancement presence has not yet been evaluated and should be performed in further studies.

As a conclusion, the evaluation of the myocardial ECV quantified by DECT in a population suspected of acute myocarditis demonstrated good diagnostic performances which allows us to consider ECV as a reliable DECT biomarker for the discrimination of myocarditis.

## Figures and Tables

**Table 1:** Baseline characteristics of the study population.

Groups	Myocarditis Group			Control Group			
Variables	*n*	Mean ± MSE	Median (Min–Max)	*n*	Mean ± MSE	Median (Min–Max)	*p*
Sex	60	49M, 11F		18	11M, 7F		0.602
Age (years)	60	32.9 ± 1.4	29.8 (18.0; 73.6)	18	35.1 ± 3.6	33.0 (15.0; 68.2)	0.731
Weight (kg)	60	75.0 ± 1.7	74.0 (50.0; 110.0)	18	73.8 ± 4.2	72.5 (39.0; 106.0)	0.606
Height (cm)	60	173.4 ± 1.0	173.0 (158; 194)	18	171.4 ± 2.3	172.5 (150; 185)	0.622
BMI (kg/m2)	60	24.9 ± 0.5	24.2 (17.2; 38.4)	18	24.9 ± 1.1	24.9 (17.3; 33.9)	0.962
LVEF (%)	60	57.6 ± 1.0	60.0 (42.0; 74.0)	18	64.2 ± 1.7	66.0 (55.0; 78.0)	0.006 *
Creatinine (µmol/L)	10	73.1 ± 4.1	75.0 (50.0; 88.0)	18	75.4 ± 3.8	72.0 (52.0; 111.0)	0.885
Troponins (ng/L)	60	8630.3 ± 1585.9	5365.0 (36.0; 62,929.0)	18	822.5 ± 339.9	214.5 (5.0; 5159.0)	0.001 *
BNP (ng/L)	44	137.4 ± 45.4	46.5 (0.1; 1700.0)	16	140.6 ± 76.0	35.0 (0.1; 1018.0)	0.303
Hematocrit (%)	60	42.2 ± 0.5	42.2 (33.3; 54.0)	18	42.7 ± 1.0	42.8 (35.4; 49.8)	0.589

MG = myocarditis group; CG = control group; MSE = mean standard error; BMI = body mass index; LVEF = left ventricular ejection fraction; BNP = brain natriuretic peptide; *p* * = significant differences (Mann–Whitney non-parametric test).

**Table 2:** Statistics of the ECV for both groups.

	ECV for MG (*n* = 60)	ECV for CG (*n* = 18)
Mean	34.18 *	30.04
Mean Standard Error	0.43	0.53
Median	34.61	29.93
Minimum	27.10	24.99
Maximum	40.54	33.06
25% Percentile	32.12	28.97
50% Percentile	34.61	29.93
75% Percentile	36.39	31.73

ECV = extracellular volume; MG = myocarditis group; CG = control group. Data are expressed in percentage. * Significant differences MG vs.CG (Mann–Whitney non-parametric test): *p* < 0.001.

**Table 3:** Spearman correlation statistics between mean ECV of each group and other parameters.

Parameters		ECV MG			ECV CG	
	*n*	Rho Spearman	*p*	*n*	Rho Spearman	*p*
Weight	60	−0.033	0.803	18	−0.423	0.080
Height	60	0.016	0.903	18	−0.362	0.140
BMI	60	−0.078	0.552	18	−0.311	0.210
Troponins	60	0.408	0.001 *	18	−0.169	0.504
LVEF	60	−0.199	0.128	18	−0.057	0.822
BNP	44	0.455	0.002 *	16	0.035	0.896

ECV = extracellular volume; MG = myocarditis group; CG = control group; *n* = number of observations; *p* = significant difference coefficient; BMI = body mass index; LVEF = left ventricular ejection fraction; BNP = brain natriuretic peptide; * = significant correlations.
